# Regulation mechanism of *Rosa roxburghii* Tratt. (*Cili*) fruit vinegar on non-alcoholic fatty liver disease

**DOI:** 10.3389/fnut.2025.1617931

**Published:** 2025-08-12

**Authors:** Yu Wang, Rui Lang, Lilang Li, Yonglan Wen, Ming Gao, Jie Zhang, Juan Yang, Qiji Li, Li Wang, Xiaosheng Yang, Xiaolong Wang, Chunzhi Xie

**Affiliations:** ^1^State Key Laboratory of Discovery and Utilization of Functional Components in Traditional Chinese Medicine, Guizhou Medical University, Guiyang, China; ^2^Natural Products Research Center of Guizhou Province, Guiyang, China; ^3^China National Research Institute of Food & Fermentation Industries Co., Ltd., Beijing, China; ^4^College of Food and Biotechnology Engineering, Xuzhou University of Technology, Xuzhou, China

**Keywords:** *Rosa roxburghii* Tratt., fruit vinegar, non-alcoholic fatty liver disease, intestinal microbiota, metabolomics, AMPK signal pathway

## Abstract

**Background:**

Non-alcoholic fatty liver disease (NAFLD) is a chronic liver disease characterized by the excessive accumulation of lipids as a pathological feature. Previous studies have demonstrated that Rosa roxburghii Tratt. fruit vinegar (RFV) played an important role in intervening in obesity and related complications by regulating the intestinal microbiota in high-fat diet mice.

**Methods:**

This study investigated the mechanisms by which RFV improves NAFLD from multiple perspectives. Potential targets were predicted by network pharmacology and molecular docking analyses. Intestinal microbial communities were detected and analyzed using 16S rRNA gene sequencing technology. Liver metabolites were detected and analyzed using ultra high performance liquid chromatography quadrupole-exactive high field-X mass spectrometer (UHPLC-Q-Exactive HF-X) and Progenesis QI software. Hepatic protein expression levels were detected and quantified using Western blotting analysis and gray-value analysis, respectively.

**Results:**

The results indicated that, RFV could improve the diversity of intestinal microbiota in NAFLD mice, reduce the ratio of Firmicutes to Bacteroidetes (F/B), and reverse the relative abundance of differential bacteria genera related to lipid accumulation and energy metabolism. The intestinal microbiota was correlated with the levels of lipid metabolism and oxidative stress in the serum and liver of mice with NAFLD. The primary bacteria genera involved were *Allobaculum, Faecalibaculum, Dubosiella, Blautia, and unclassified_f_Lachnospiraceae*. A total of 441 liver metabolites were identified in NAFLD mice and participating in 21 metabolic pathways. Glycerophospholipid metabolism may be an important pathway regulating NAFLD by RFV. Phosphatidylcholines (PC) and lysophosphatidylcholinergic (LPC) metabolites were significantly regulated by RFV and had significant correlation with differential microbiota. RFV may improve NAFLD by regulating lipid synthesis in the adenosine 5’-monophosphate (AMP)-activated protein kinase (AMPK) pathway. Western blotting analysis showed that, RFV could activate the AMPK phosphorylation, and reduce the expression of fatty acid synthase (FASN) and sterol regulatory element-binding protein 1 (SREBP-1c), resulting in the inhibition of fatty acids de novo synthesis and lipid accumulation.

**Conclusion:**

As a functional food, RFV has been proven to be effective in improving NAFLD. The underlying mechanisms involve the modulation of the intestinal microbiota and metabolites balance, and regulation on lipid disorders through AMPK signaling pathway.

## 1 Introduction

*Rosa roxburghii* Tratt., also known as *Cili*, is a member of the genus *Rosa* and family *Rosaceae*. It has high edible and medicinal values as a source of homologous medicine and food in China (1, 2). It is rich in vitamin C (3), polyphenols (4), triterpenoids (5), superoxide dismutase (6), polysaccharide, etc. (7), and has the effects of anti-oxidation (8), hypolipidemia (9), hypoglycemia (10), anti-aging, etc. (11). However, the sour and astringent taste of *Rosa roxburghii* Tratt. products in the market limit their promotion and development. Numerous studies have demonstrated that probiotic fermentation can effectively remove or reduce anti-nutritional factors, such as tannins and phytic acid in raw materials, producing foods with higher nutritional value (12–14). *Rosa roxburghii* Tratt. fruit vinegar (RFV) was fermented using a mixture of *Lactobacillus plantarum*, *Acetobacter pasteurianus*, and *Saccharomyces cerevisiae*. The sour and astringent taste of *Rosa roxburghii* Tratt. was significantly improved by this fermentation method. Vinegar is a fermented product produced through alcohol fermentation followed by acetic acid fermentation. Studies have demonstrated that vinegar can improve hypertension, hyperlipidemia, obesity, and diabetes and regulate intestinal microbiota (15–17). Mohamad et al. (18) reported that the gavage of obese mice using coconut water vinegar augmented the abundance of *Akkermansia* and Bacteroides. Similarly, Hosoda et al. (19) found that ginkgo vinegar inhibited high fat diet-induced weight gain in mice and reduced the size of adipocytes. Obesity is associated with many chronic diseases such as atherosclerosis, cardiovascular disease, inflammatory bowel disease, hyperlipidemia, and non-alcoholic fatty liver disease (NAFLD). Previous studies have demonstrated that RFV plays an important role in intervening in obesity and related complications by regulating the intestinal microbiota in high-fat diet mice (20). Consequently, we hypothesize that RFV, a type of fruit-fermented vinegar, may ameliorate obesity-induced NAFLD.

NAFLD is a metabolic disorder caused by a high-fat diet. In recent years, the prevalence of NAFLD has continued to rise, affecting nearly one billion individuals globally. Approximately 40% of patients with NAFLD progress to severe non-alcoholic steatohepatitis (NASH) (21). The pathogenesis of NAFLD is complex. The widely accepted “multiple hits” hypothesis posits that various factors collectively contribute to the onset of the disease, including lipid accumulation, oxidative stress, endoplasmic reticulum stress, and lipotoxicity, all of which are implicated in NAFLD development (22). These factors can influence the fat content of hepatocytes and the inflammatory environment of the liver, leading to chronic liver inflammation (23). Due to the complex composition of the RFV, the specific mechanism by which it improved NAFLD was not yet clear.

Recently, numerous studies have demonstrated that an imbalance in intestinal microbiota may lead to metabolic disorders such as obesity, diabetes, metabolic syndrome, and cardiovascular disease. Studies have demonstrated that the “gut–liver” axis pathway plays a crucial role in regulating liver disease through the intestinal microbiota and its metabolites (24). The occurrence and development of NAFLD are often accompanied by a steady-state imbalance in the intestinal microbiota (25). The liver is the primary source of endogenous metabolites, precursors of signaling molecules, and enzymes involved in detoxification. Therefore, the liver metabolome comprises dynamic and complex small-molecule metabolites (26). Through metabolomics analysis, Udan Lipid-reducing prescription was found to reverse lipid metabolism disorders caused by a high-fat diet and improve hepatocyte steatosis by downregulating arachidonic acid, phosphatidylethanolamine (PE), and triglyceride (TG) and upregulating acylcarnitine (27). AMPK is an important metabolic sensor that can regulate the energy homeostasis of cells (28), and its activity is suppressed in energy surplus. Studies have demonstrated that AMPK inhibition may stimulate anabolic pathways (lipid synthesis) and attenuate the catabolic pathway (*β*-oxidation). Furthermore, it aggravates the degeneration of adipocytes, liver injury, and liver fibrosis and subsequently accelerates the transformation of NASH to cirrhosis and hepatocellular carcinoma (29, 30). Consequently, AMPK activity could be used as a target to regulate NAFLD.

In this study, we systematically explored the regulatory mechanism of RFV in NAFLD mice. UHPLC-Q-Exactive HF-X, network pharmacological methods, and molecular docking were used to predict the targets. Illumina MiSeq high-throughput sequencing, non-targeted liquid chromatography–mass spectrometry (LC–MS) metabolomics, and Western blotting analysis were used to analyze fecal intestinal microbiota, liver metabolites, and liver protein expression, respectively.

## 2 Materials and methods

### 2.1 Materials and reagents

*Rosa roxburghii* Tratt. fruits were obtained from Guizhou Saisi *Rosa roxburghii* Tratt Health Industry Co., Ltd. (Qiannan, Guizhou, China). A DNA extraction kit was purchased from Omega Bio-tek Co., Ltd. (Omega Bio-tek, Norcross, GA, USA). The library building kit was procured from Bioo Scientific Co., Ltd. (Bioo Scientific, Norcross, GA, USA). A sequencing kit was obtained from Illumina Co., Ltd. (Illumina, Norcross, GA, USA). The bicinchoninic acid (BCA) protein concentration determination kit was received from Beyotime Biological Reagent Company (Guangxi, China). Polyvinylidene fluoride membrane (PDVF) was sourced from Millipore Co., Ltd. (Millipore, Bedford, MA). AMPK monoclonal antibody, AMPK phosphorylated monoclonal antibody P-AMPK monoclonal antibody, SREBP-1c monoclonal antibody, FASN monoclonal antibody, and *β*-actin monoclonal antibody were supplied by Abcam Co., Ltd. (Abcam, Cambridge, USA). Horseradish peroxidase (HRP)-labeled goat anti-rabbit/mouse IgG secondary antibody was provided by Servicebio Co., Ltd. (Wuhan, China).

### 2.2 Preparation of RFV

RFV was prepared as previously described (31). In brief, the fresh *Rosa roxburghii* Tratt. fruits were cleaned and beaten thoroughly sequentially, and water was added at a ratio of 1:3 (g:mL). The *Rosa roxburghii* Tratt. juice (RRJ) was obtained. The mixed fermentation starter (*Lactobacillus plantarum*, *Acetobacter pasteurianus*, and *Saccharomyces cerevisiae* at a ratio of 1:1:1) was activated with warm water and added at a mass ratio of 0.02%. Finally, at a constant temperature of 25–27°C, the fermentation broth was supplemented with 10% (*m/m*) sucrose for aerobic fermentation for up to 20 days. The fermentation broth was stirred every 3 days. The fermentation was completed with a solid content of 7–8%, and a pH value of approximately 4. RFV was obtained by filtration and used for subsequent experiments.

### 2.3 Network pharmacology analysis based on active compounds in RFV

UPLC–MS was used to identify the compounds of RFV. The specific method has been previously described (32). According to the previous experimental results, 70 compounds were identified in RFV through UPLC–MS analysis, including 27 phenolic compounds, 14 terpenoids, 5 organic acids, 4 sugars, 3 lactones, 3 amino acids, 2 lipids, 1 vitamin, and 11 other compounds (32). Based on the active compounds in RFV, network pharmacology methods were employed to predict their targets. In brief, the network pharmacology methods primarily included the following steps: first, potential target genes for the compounds in RFV and NAFLD were obtained through the Swiss Target Prediction^1^ and GeneCards databases (see text Footnote 1), respectively. The intersecting targets were screened using the Venny 2.1 online tool.^2^ The intersecting targets were then imported into the STRING database to construct a protein–protein interaction (PPI) network.^3^ Cytoscape software was used for visualization. The CytoNCA plugin was used to select and visualize core targets. Subsequently, gene ontology (GO) and Kyoto encyclopedia of genes and genomes (KEGG) analyses were performed using the DAVID database.^4^ The results were visualized using the Wei Sheng Xin Cloud Platform.^5^ Finally, the compounds, targets, and pathways were integrated to construct a network relationship diagram, revealing the mechanism by which RFV improves NAFLD.

### 2.4 Molecular docking

The spatial data files (SDF format) of the active compounds were retrieved from the PubChem database.^6^ Three-dimensional structures of the core target proteins were obtained from the protein data bank (PDB, https://www.rcsb.org/). Active compounds and target proteins were prepared through dehydration, hydrogenation, and structural refinement using PyMOL. PyMOL software was utilized to visualize and analyze the protein–ligand interactions. Binding energy values were used to evaluate molecular docking results.

### 2.5 Animal experiment

Experimental grouping: Animal studies were performed in accordance with the Chinese regulations on the management of laboratory animals (State Council Decree No. 676, 2017) and the animal research: reporting of *in vivo* experiments (ARRIVE) guidelines. All protocols were approved by the Animal Ethics Committee of Guizhou Medical University (SYXK (Gui) 2023–0002). Forty C57BL/6 J male mice (8-week-old, 20–22 g) were obtained from Beijing Xiao Shu You Tai Biotechnology Co., Ltd. (Beijing, China). These mice were housed in a standard animal facility maintained at 23–25°C with 55–60% humidity. They underwent an adaptive feeding period for 1 week. Subsequently, 40 mice were randomly divided into five groups (*n* = 8): a normal diet (Control) group, a high-fat diet (NAFLD) group, an RRJ group, a low-dose RFV (RFV-L) group, and a high-dose RFV (RFV-H) group.

One week later, a NAFLD model was established based on the method described in this reference (33). Except for the Control group, the mice in the other groups were fed a high-fat diet for 12 weeks. During the modeling period, mice in the RRJ, low, and high-dose RFV groups were intragastrically administered with RRJ and RFV, respectively. The Control and NAFLD group received saline. All treatments were administered at 0.01 mL/10 g body weight, during which the body weight of mice was recorded every 5 days. Based on the previous studies (34) and results of our pre-experiments, RRJ was diluted 2-fold (50.0%, v/v), and RFV was diluted 8-fold and 4-fold to prepare as low-dose RFV (12.5%, v/v) and high-dose RFV (25.0%, v/v), respectively.

### 2.6 16S rRNA gene sequencing of intestinal microbiota

Total DNA was extracted using the E.Z.N.A.® Soil DNA Kit (Omega Bio-tek, Norcross, GA, USA). The concentration and purity were determined using NanoDrop 2000 (Thermo Fisher Scientific, San Jose, CA, USA). DNA integrity was assessed using 1% agarose gel electrophoresis at a voltage of 5 V/cm for 20 min (Biowest Co., Ltd., Spain). The Illumina MiSeq platform (Microeco Tech Co., Ltd., Shenzhen, China) was used to amplify and sequence the highly variable region of the 16S rRNA gene V3–V4 using the following primers: 338F: ACTCCTACGGGAGGCAGCAG and 806R: GGACTACHVGGGTWTCTAAT.

### 2.7 Liver metabolites detection by non-targeted LC-MS

Chromatographic conditions: The chromatographic column was ACQUITY UPLC® HSS T3 (100 × 2.1 mm, 1.8 μm; Waters, Milford, USA). The parameters were set as follows: mobile phase A consisted of 95% water and 5% acetonitrile (containing 0.1% formic acid), and mobile phase B consisted of 47.5% acetonitrile, 47.5% isopropyl alcohol, and 5% water (containing 0.1% formic acid). The injection volume was 3 μL, and the column temperature was 40°C.

Mass spectral conditions: Electrospray was used to ionize the sample. The spectra were obtained using electrospray ionization with both positive and negative ionization modes, using a Q-Exactive Focus (Thermo Scientific, Waltham, MA, USA). The parameters were set as follows: The negative ion spray voltage and positive ion spray voltage were set to 3.50 kV, the sheath gas was 50 arb, the auxiliary gas was 13 arb, the scanning range was 70–1,050, the capillary temperature was 325°C, the resolution was 3,500, and collision voltage was set to 40 eV.

### 2.8 Western blotting analysis

The expression levels of AMPK, P-AMPK, SREBP-1c, and FASN in the livers of each group were detected by Western blotting. Proteins were extracted from the protein lysate and denatured at 100°C for 10 min. Protein samples were subjected to sodium dodecyl sulfate-polyacrylamide gel electrophoresis (SDS-PAGE), and the proteins were transferred to polyvinylidene fluoride (PVDF) membranes, which were then blocked with a 5% skimmed milk solution. Subsequently, the membranes were incubated overnight at 4°C with rabbit anti-AMPK, rabbit anti-P-AMPK, rabbit anti-SREBP-1c, rabbit anti-FASN, and rabbit anti-*β*-actin antibodies at a dilution of 1:1000. Finally, the membranes were incubated with the corresponding anti-mouse secondary antibody (1:3000) at room temperature for 1 h. The signal was detected using a chemiluminescent imaging system (XRS; Bio-Rad, Fort Hershey, USA). Protein expression was determined by grayscale analysis of the target protein bands using ImageJ software. The relative expression levels of SREBP-1c and FASN were expressed as the gray ratio of SREBP-1c, FASN, and β-actin, and the relative expression level of P-AMPK was expressed as the gray ratio of P-AMPK and AMPK.

### 2.9 Statistical analysis

GraphPad Prism (version 5) and Origin (version 2022) software were used for mapping analysis. The *t*-test was used for comparisons between two groups. If *p* < 0.05, the difference between the two groups was considered statistically significant.

## 3 Results

### 3.1 Prediction of potential targets based on active compounds in RFV

**Figure 1:**
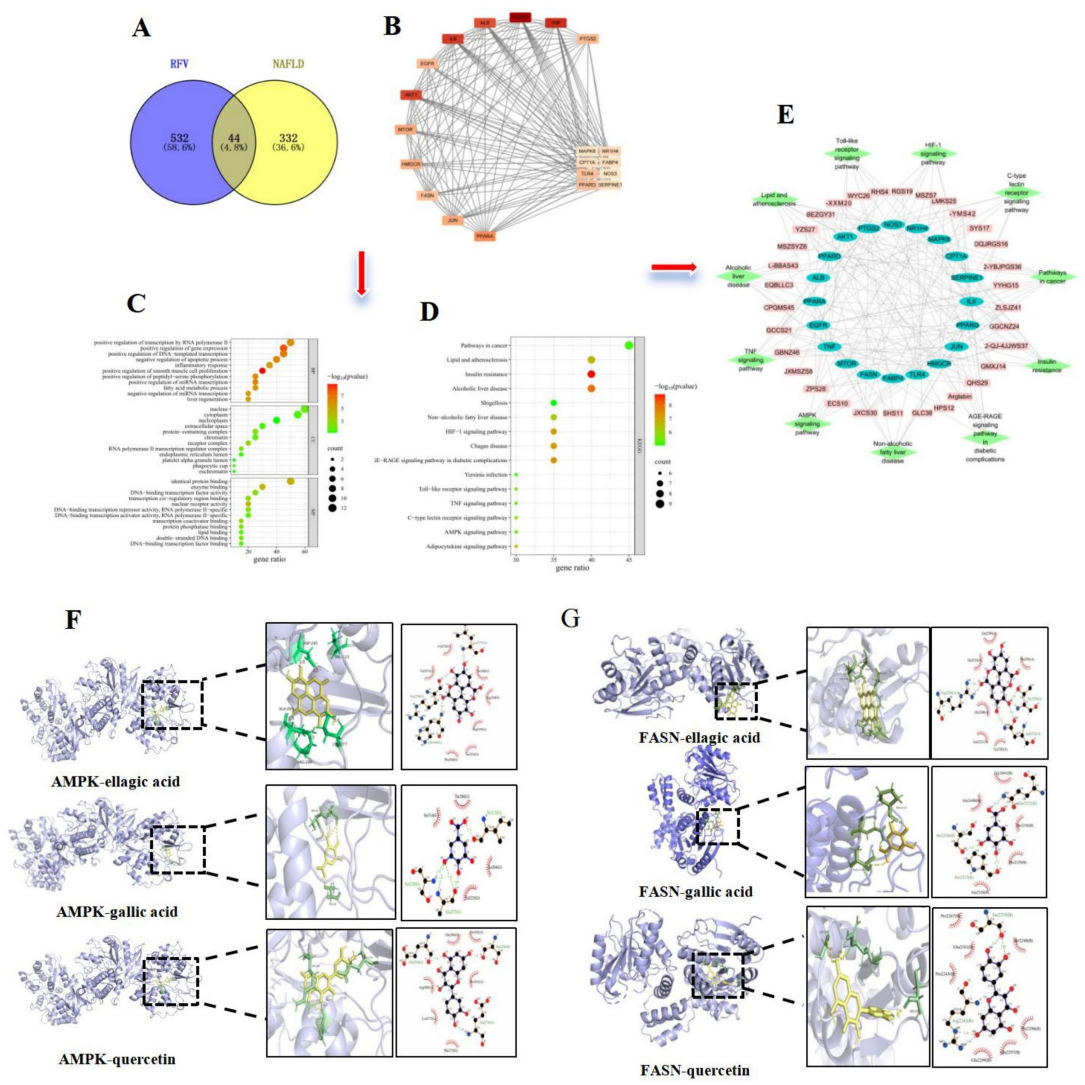
Results of network pharmacology and molecular docking. Venn diagram of potential targets of RFV on NAFLD **(A)**; PPI network diagram of key targets **(B)**; GO function enrichment analysis of potential targets **(C)**; KEGG pathway enrichment of potential targets **(D)**; Compound-key target-pathway visualization network **(E)**; The molecular docking diagram of AMPK and ellagic acid, gallic acid, quercetin. AMPK-ellagic acid (−9.1 kcal/mol), gallic acid (−5.9 kcal/mol), and quercetin (−8.7 kcal/mol) **(F)**; The molecular docking diagram of FASN and ellagic acid, gallic acid, quercetin. FASN-ellagic acid (−8.2 kcal/mol), gallic acid (−6.6 kcal/mol), and quercetin (−8.4 kcal/mol) **(G)**.

The compounds in RFV were identified as previously described. A total of 70 compounds were identified in RFV through UPLC-MS analysis, including 27 phenolic compounds, 14 terpenoids, 5 organic acids, 4 sugars, 3 lactones, 3 amino acids, 2 lipids, 1 vitamin, and 11 other compounds. Based on these findings, network pharmacology was used to predict the targets and pathways by which RFV regulates NAFLD. A total of 1,816 potential targets of RFV were identified using the ITCM database (with a probability > 0). Simultaneously, 1,399 targets associated with NAFLD were identified using the GeneCards database. Among 1,399 targets, 377 with a relevance score > 0.88 (twice the median relevance score) were selected for further analysis. A Venn diagram indicated that 44 common targets were identified across these datasets (Figure 1A). Using the CytoNCA plugin, a PPI network was constructed for the top 20 target genes based on their degree (Figure 1B). Protein nodes, such as PPARG, TNF, IL6, AKT1, ALB, and FASN, exhibited higher connectivity, indicating their central roles within the network.

### 3.2 Prediction of potential pathways based on targets

The 20 intersecting targets were analyzed for GO functional annotation and KEGG pathway using the DAVID (version 6.8.0) database. The results were visualized on the Wei Sheng Xin Cloud Platform. GO results indicated that biological process (BP) included inflammatory responses, fatty acid metabolism, and liver regeneration (Figure 1C). The proteins encoded by the intersecting genes were primarily located in the nucleus, cytoplasm, and nucleoplasm. Molecular function (MF) primarily involves the binding of proteins, enzymes, and lipids. The results of KEGG pathway analysis are illustrated in Figure 1D. The following 11 relevant pathways were selected: lipid and atherosclerosis pathway, AGE-RAGE signaling pathway, AMPK pathway, C-type lectin receptor pathway, insulin resistance, HIF − 1 signaling pathway, toll-like receptor signaling pathway, TNF signaling pathway, alcoholic liver disease, NAFLD, and pathways in cancer. A compound-gene-pathway network diagram was created to visually illustrate the intricate relationships among compounds, targets, and pathways (Figure 1E). The lines in the diagram reflect the relationships among compounds, targets, and pathways. The network diagram further revealed that the active compounds of RFV might improve NAFLD through multiple targets and pathways.

### 3.3 Molecular docking

Molecular docking results revealed that the key active compounds (quercetin, ellagic acid, and gallic acid) were molecularly docked with core targets associated with the AMPK pathway (AMPK and FASN). Molecular docking results indicated that all three compounds could dock with AMPK and FASN proteins (Figures 1F,G). They all exhibited binding energies of <−5 kcal/mol, indicating a strong binding affinity. The AMPK pathway may be an important target in NAFLD regulation. This was experimentally validated below.

### 3.4 Effect of RFV on intestinal microbiota in NAFLD mice

**Figure 2:**
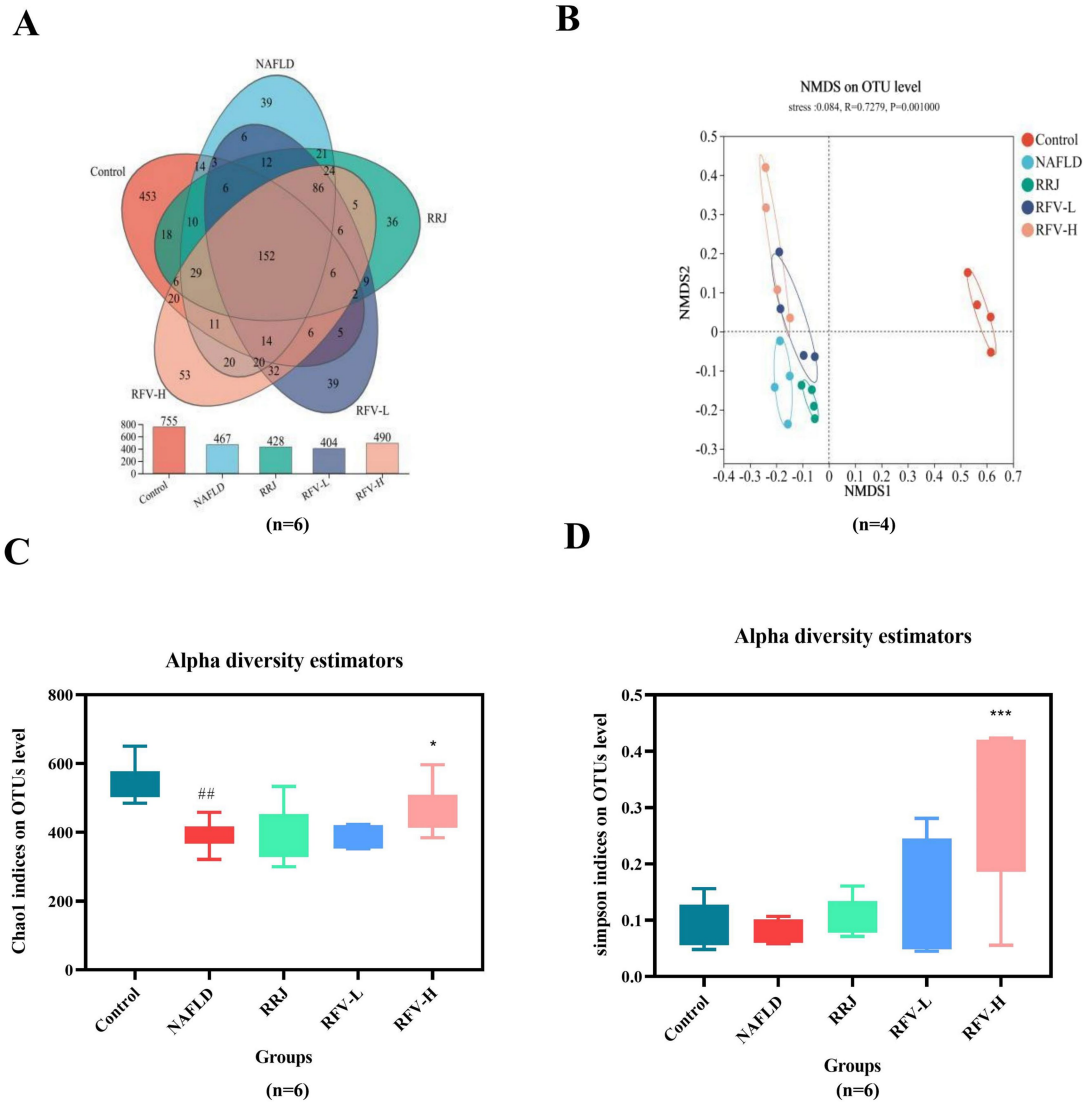
Effect of RFV on intestinal microbiota in NAFLD mice. Venn diagram of OTUs **(A)**; NMDS analysis of β diversity **(B)**; Chao1 indices of Alpha diversity **(C)**; Simpson indices of Alpha diversity **(D)**. The Control, NAFLD, RRJ, RFV-L, and RFV-H were normol diet group, high-fat diet group, *Rosa roxburghii* juice, low dosage *Rosa roxburghii* fruit vinegar group, and high dosage *Rosa roxburghii* fruit group, respectively. ^#^*p*<0.05, compared with Control group, ^*^p<0.05, compared with NAFLD group (*n*=6).

The samples were analyzed using Illumina MiSeq high-throughput sequencing to investigate the effect of RFV on the intestinal microbiota composition (*n* = 6). The Venn diagram illustrates the total and overlapping numbers of operational taxonomic units (OTUs) in each group (Figure 2A). Control, NAFLD, RRJ, RFV-L, and RFV-H groups had 755, 465, 428, 404, and 490 OTUs, respectively. All five groups shared 152 OTUs, indicating the presence of at least 152 symbiotic bacteria common to each group. Furthermore, the data revealed that the Control group had 453 unique OTUs, the NAFLD group had 39 unique OTUs, and RFV-L and RFV-H groups had 39 and 53 unique OTUs, respectively. Alpha diversity estimation analyses revealed that the Chao1 indices (391) in the NAFLD group decreased significantly (*p* < 0.001) compared to the Control group, whereas the Simpson indices (0.08) did not indicate any significant change. After administration of high-dose RFV, Chao1 indices (465) and Simpson indices (0.30) increased significantly (*p* < 0.05, *p* < 0.001) (Figures 2C,D).

**Figure 3:**
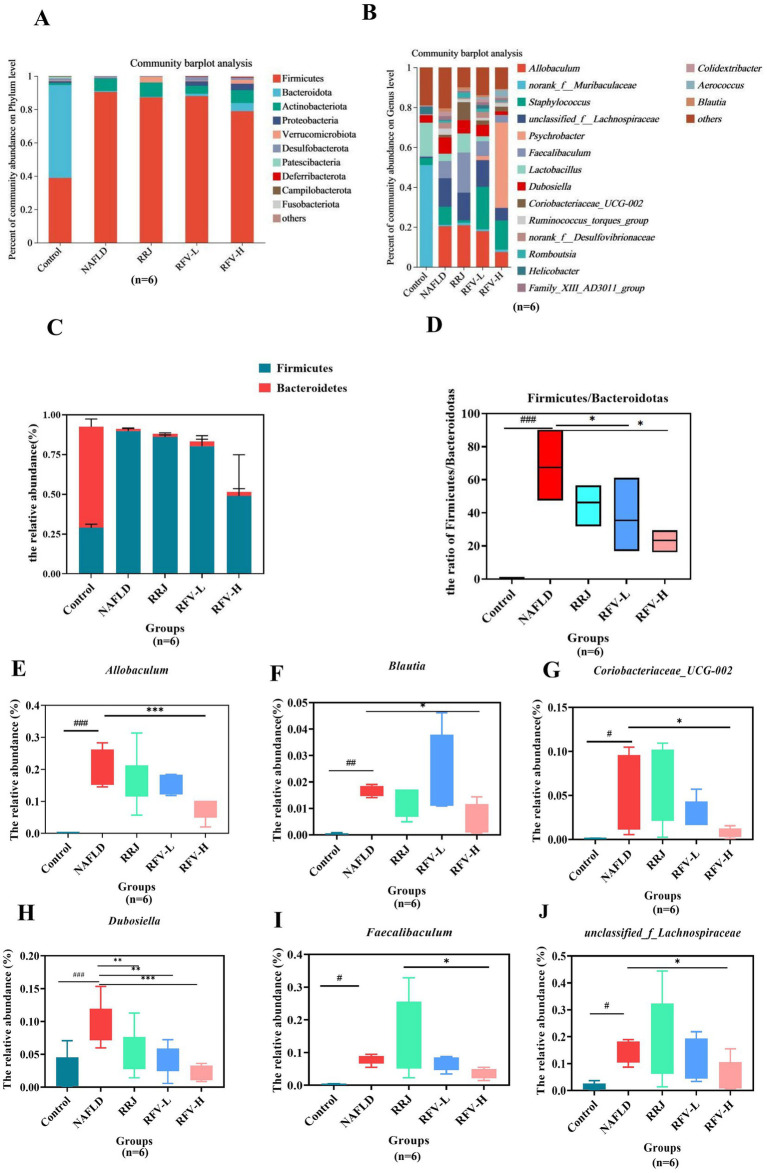
Species composition and bacteria with significant differences in relative abundanceat at the phylum and genus level. Histogram of relative abundance at phylum level **(A)**; Histogram of relative abundance at genus level **(B)**. Relative abundance of Firmicutes and Bacteroidetes **(C)**; Ratio of F to B (F/B) **(D)**; The relative abundance at genus level of *Allobaculum* **(E)**; *Blautia* **(F)**; *Coriobacteriaceae_UCG-002* **(G)**; *Dubosiella* **(H)**; *Faecalibaculum* **(I)**; *Unclassified_f_Lachnospiraceae* **(J)**. ^#^*p*<0.05, compared with Control group, ^*^p<0.05, compared with NAFLD group.

The microbial composition significantly changed after HFD and RFV intervention. There was a clear separation between the Control and NAFLD groups. Each administration group was significantly different from the NAFLD group (Figure 2B). The above results indicate that an HFD can alter the structure of the intestinal microbiota in mice, and RFV can improve the situation to a certain extent; therefore, the microbiota structure is closer to that of the Control group. The composition of the intestinal microbiota was analyzed at the phylum and genus levels (Figures 3A,B) and (Supplementary Figures 1A,B). At the phylum level, Firmicutes, Bacteroidetes, and Actinobacteria were dominant in each group. The relative abundances of Firmicutes in Control, NAFLD, RRJ, RFV-L, and RFV-H groups were 55.71, 0.57, 0.32, 1.44, and 4.86%, respectively. The relative abundances of Bacteroidetes were 55.71, 0.57, 0.32, 1.44, and 4.86%, respectively. The NAFLD group exhibited a significant increase in Firmicutes and a significant decrease in Bacteroidetes compared to the Control group (*p* < 0.001) (Figure 3C). As a result, the ratio of Firmicutes to Bacteroidetes (F/B) was highest in the NAFLD group. After the administration of high-dose RFV, the relative abundance of Firmicutes decreased significantly (*p* < 0.01), Bacteroidetes increased, and F/B decreased significantly (*p* < 0.01) (Figure 3D), indicating that RFV could improve the imbalance in microbial abundance caused by NAFLD, and the community composition was reversed to be similar to that of the Control group. Compared to the Control group, the relative abundance of *Allobacterium*, *Faecalibaculum*, *Coriobacteriaceae_UCG-002*, *Dubosiella*, *Blautia*, and *unclassified_f_Lachnospiraceae* increased in the NAFLD group (*p* < 0.05). Compared to the NAFLD group, RFV-H significantly reduced the relative abundance of A*llobaculum*, *Faecalibaculum*, *Dubosiella*, *Blautia*, *Coriobacteriaceae_UCG-002*, and *unclassified_f_Lachnospiraceae* (Figures 3E-J). Overall, RFV reversed intestinal microbiota disruption caused by NAFLD.

### 3.5 Effect of RFV on liver metabolites in NAFLD mice

**Figure 4:**
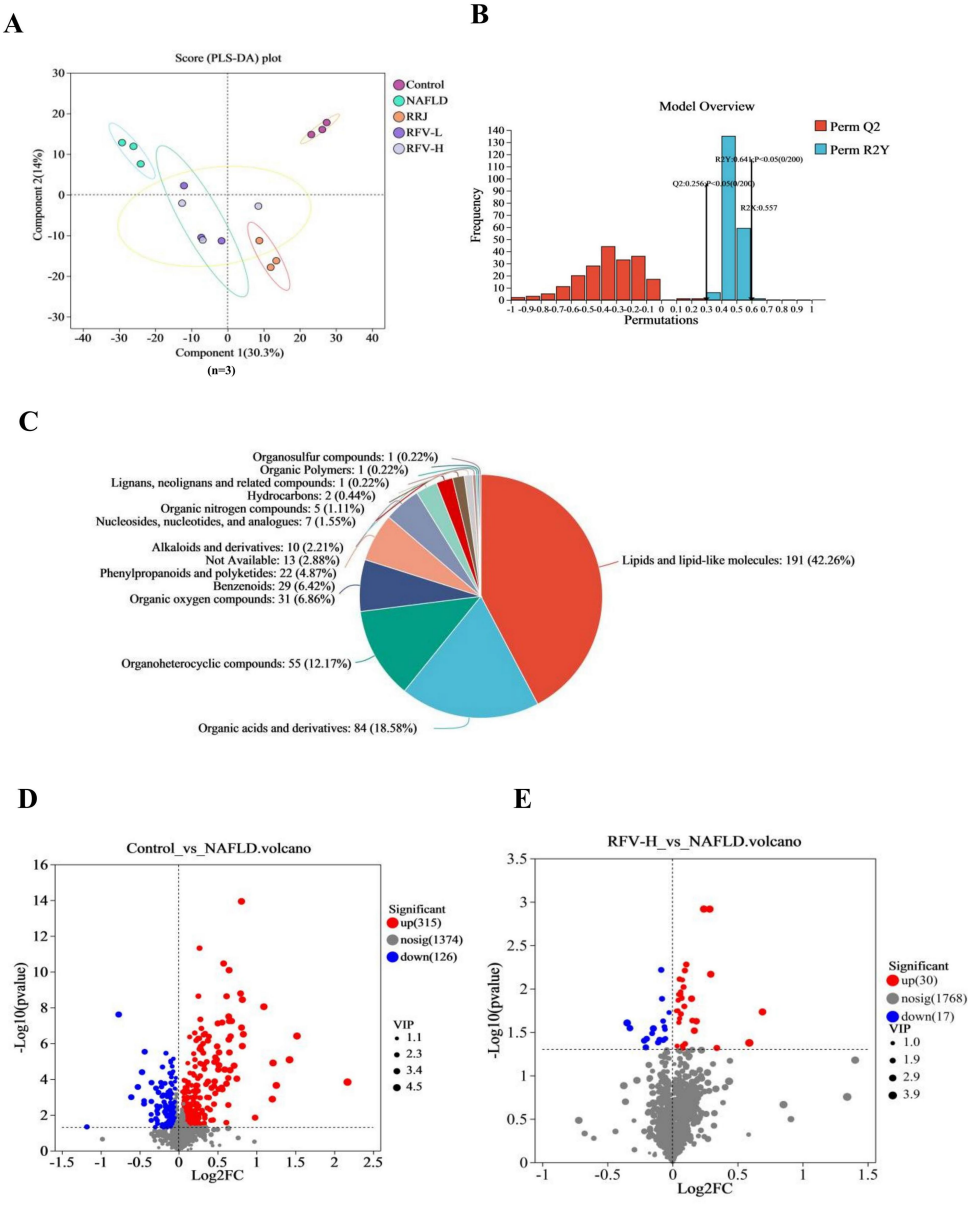
Effect of RFV on liver metabolites in NAFLD mice. PLS-DA score chart **(A)**; PLS-DA displacement test map **(B)**; Pie chart of differential metabolite classification **(C)**; The trend of metabolites change in Control *vs.* NAFLD **(D)**; The trend of metabolites change in RFV-H *vs.* NAFLD **(E)** (*n*=6).

Partial least squares discriminant analysis (PLS-DA) demonstrated that liver metabolites were significantly different due to HFD and RFV intervention. The induction of NAFLD significantly changed liver metabolites. After the administration of RRJ and RFV, the metabolite composition was different from that of the NAFLD group and closer to the Control group (Figure 4A). A replacement test was performed using the PLS-DA model (R^2^Y = 0.64, *Q*^2^ = 0.256, *p* < 0.05), indicating that the model was stable and reliable (Figure 4B).

Enrichment analysis of differential metabolites was performed using metaboAnalyst enrichment analysis. A total of 490 different metabolites were screened among the five groups (*p* < 0.05). These metabolites included 191 lipids and lipid-like molecules, 84 organic acids and their derivatives, 55 organic heterocyclic compounds, 31 benzenoid compounds, 29 organic oxygen compounds, 22 phenylpropanoids and polyketones, 10 nucleosides, nucleotides, and analogs, 7 alkaloids and their derivatives, 5 organic nitrogen compounds, 5 organic sulfur compounds, 2 hydrocarbon derivatives, 1 hydrocarbon compound, 1 lignin, new signaling, and related compounds, 1 organic polymer, and 13 other metabolites (Figure 4C).

There were 441 and 47 differential metabolites in the Control *versus* NAFLD group and NAFLD *versus* RFV-H group (*p* < 0.05, VIP value > 1, Fold change = 1), respectively. In both positive and negative ion modes, the volcano plot revealed that 315 metabolites were upregulated and 26 metabolites were downregulated in the Control versus NAFLD groups, whereas 30 metabolites were upregulated and 17 metabolites were downregulated in the NAFLD versus RFV-H groups (Figures 4D,E).

### 3.6 Metabolic pathway analysis

**Figure 5:**
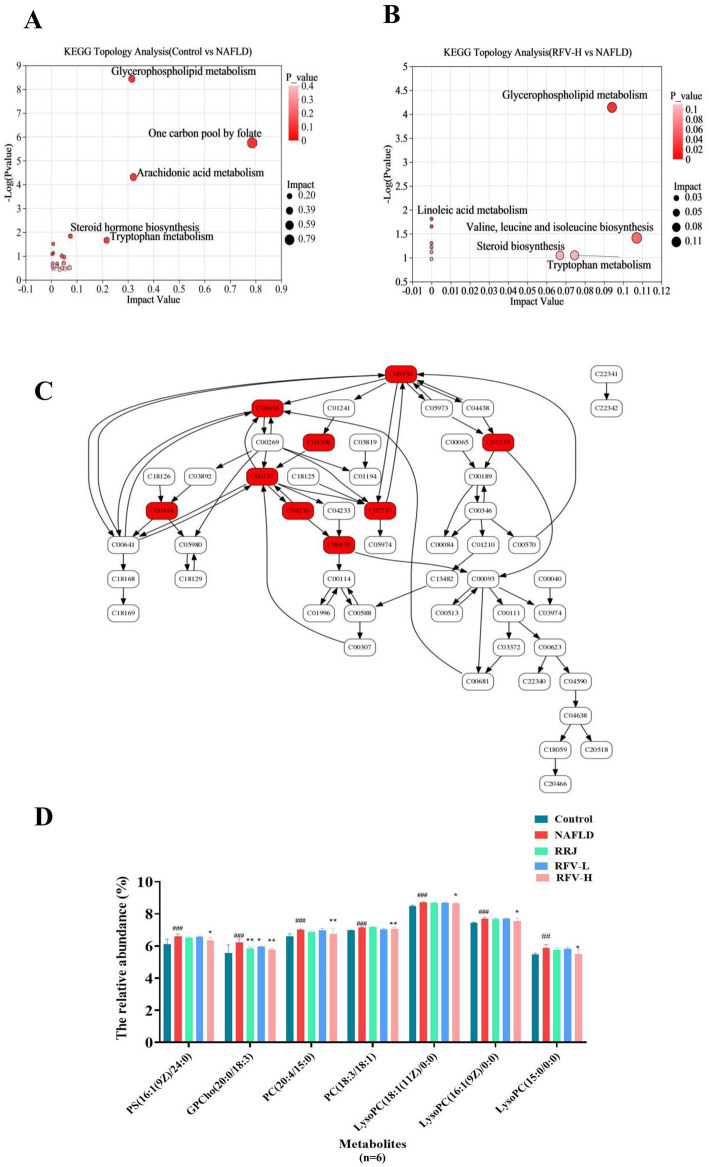
Analysis of enriched pathways of differential metabolites. Topology analysis diagram **(A,B)**; Glycerophospholipid metabolism pathway diagram **(C)**; Histogramgraph of the relative abundance of metabolites in each group **(D)**. ^#^*p*<0.05, compared with Control group, ^*^p<0.05, compared with NAFLD group. C00350: phosphatidylethanolamine (PE), C00416: phosphatidic acid (PA; 18:0/18:2 (9Z and 12Z)), C04308: PE-NMe2 (14:0/14:0), C01233: Sn-glycoro-3-Phosphatethanolamine, C0015: phosphatidylcholine (PC), C00344: glycerophosphate (PGs), C04230: lysophosphatidylcholine (LysoPC), C02737: Phosphatidylserine (PS; 16:1(9Z)/(24:0)), C00670: Glycerophosphocholine and Glycerylphosphorylcholine.

Metabolic sets were created for metabolites of the two comparison groups. KEGG topology analysis was used to examine the pathways associated with the metabolites (Figures 5A,B) and subsequently screened for key pathways based on *p*-*values* and impact values (Table 1). Except for metabolic pathways with an impact of 0, 21 metabolic pathways were involved between the Control and NAFLD groups. Arachidonic acid metabolism, glycerophospholipid metabolism, tryptophan metabolism, and the biosynthesis pathways of ubiquinone and other terpenoid quinones were the most significant. The impact values were 0.320, 0.314, 0.216, and 0.007, respectively, with *p*-values < 0.05.

**Table 1:** Analysis results of metabolic pathway of Control *vs.* NAFLD and RFV-H *vs.* NAFLD group.

Groups	Number	Description	Total	Hits	Impact	Raw *p*
Control *vs.* NAFLD	1	Arachidonic acid metabolism	37	5	0.320593269	0.0000474
	2	Glycerophospholipid metabolism	52	9	0.314681886	0.00000000355
	3	Tryptophan metabolism	56	3	0.216213498	0.021298742
	4	Ubiquinone and other terpenoid-quinone biosynthesis	65	3	0.006900359	0.030441476
RFV-H *vs.* NAFLD	1	Valine, leucine, and isoleucine biosynthesis	23	1	0.107058824	0.038646325
	2	Glycerophospholipid metabolism	52	3	0.094173045	0.000360843

In the RFV-H group, RFV-H significantly regulated NAFLD through multiple pathways, including valine, leucine, and isoleucine biosynthesis, as well as glycerophospholipid metabolism. The impact values were 0.107 and 0.094, with *p* < 0.05. The glycerophospholipid metabolic pathway was common to NAFLD and Control, RFV-H groups, suggesting that it is a potential target for RFV intervention in NAFLD. Therefore, the related metabolites are discussed in detail.

The glycerophospholipid metabolic pathway is illustrated in Figure 5C, where the red portion represents significantly different metabolites between the Control and NAFLD groups. The relative abundance of these metabolites was significantly regulated by RFV (Figure 5D) (*n* = 6). The high-fat diet significantly upregulated the levels of PCs, PS, and LysoPCs (*p* < 0.001). Following the intervention across various treatment groups, these metabolite levels varied during recovery, with RFV-H exhibiting the most significant effect (*p* < 0.01).

### 3.7 Correlation analysis between intestinal microbiota and lipid metabolism and oxidative stress indexes in NAFLD mice

**Figure 6:**
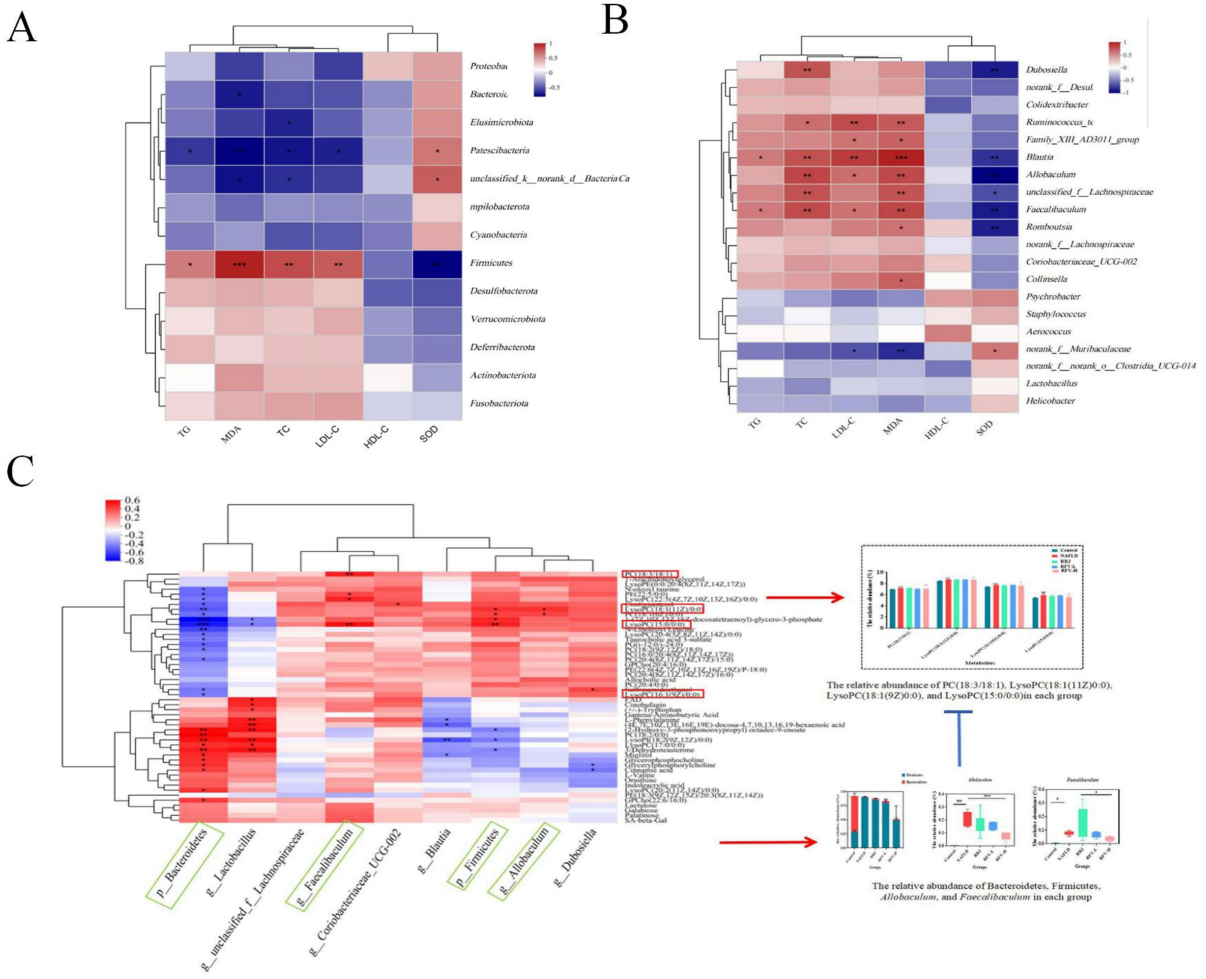
The correlation among intestinal microbiota, liver metabolites, and NAFLD-related indexes based on heatmap correlation analysis. The correlation between Bacteria at each taxonomic level and mouse lipid metabolism and oxidative stress indicators **(A,B)**; The correlation between differential metabolites and differential microflora **(C)**; *p* ≤ 0.05 is marked as *; *p* ≤ 0.01 is marked as **; *p* ≤ 0.001 is marked as ***. Blue box: dominant intestinal microbiota; Red box: significant metabolites.

To investigate the correlation between intestinal microbiota and superoxide dismutase (SOD), triglyceride (TG), cholesterol (TC), malondialdehyde (MDA), high-density lipoprotein (HDL-C), and low-density lipoprotein (LDL-C) in NAFLD mice, species were selected for correlation network analysis based on Spearman’s correlation, |r| > 0.6, *p* < 0.05 (Figures 6A,B). The heatmap of the correlation analysis demonstrated that Firmicutes exhibited a significant positive correlation with MDA, TC, LDL-C, and TG and a negative correlation with SOD. Patescibacteria was negatively correlated with MDA (*p* < 0.05), TC, LDL-C, and TG (*p* < 0.05) and positively correlated with SOD (*p* < 0.05). Bacteroidetes were negatively correlated with MDA (*p* < 0.001), and *Unclassifiedk_norank_d_Bacteria* were positively correlated with SOD (*p* < 0.05). TC and LDL-C exhibited a positive correlation with *Allobaculum*, *Faecalibaculum* (*p* < 0.01, *p* < 0.05), and *Blautia* (*p* < 0.01). TC was positively correlated with *Dubosiella* (*p* < 0.01), whereas LDL-C was negatively correlated with *norank_f_Muribaculaceae* (*p* < 0.05). MDA levels were positively correlated with *Collinsella*, *Romboutsia, Family_XIII_AD3011_group* (*p* < 0.05), *Faecalibaculum*, *unclassified_f_Lachnospiraceae*, *Allobaculum*, *Ruminococcus_torques_group* (*p* < 0.01), *and Blautia* (*p* < 0.001), whereas they were negatively correlated with *norank_f_Muribaculaceae* (*p* < 0.01). SOD exhibited a negative correlation with *Romboutsia*, *Faecalibaculum*, *Blautia* (*p* < 0.01), *unclassified_f_Lachnospiraceae* (*p* < 0.05), and *Allobaculum* (*p* < 0.001) and a positive correlation with *norank_f_ Muribaculaceae* (*p* < 0.05). Consequently, a correlation was found between the intestinal microbiota, lipid metabolism, and oxidative stress levels in mice with NAFLD. The main bacterial genera were *Allobaculum*, *Faecalibaculum*, *Dubosiella*, *Blautia*, *unclassified_f_Lachnospiraceae*, *and norank_f_ Muribaculaceae.*

### 3.8 Correlation analysis between differential metabolites and intestinal microbiota in NAFLD mice

Spearman was used to analyze the correlation between the related bacteria and the differential metabolites affected by RFV (Figure 6C). Glycerophospholipids such as phosphatidylethanolamine: PE (22:5/0:0), phosphatidylcholine: PC (18:1(6Z)/0:0), PC (22:5/0:0), PC (18:2(9Z, 12Z)/18:0), PC (16:0/20:4(8Z, 11Z, 14Z, 17Z)), PC (20:4(8Z, 11Z, 14Z, 17Z)/15:0), lysophosphatidylcholine: LysoPC (22:5(4Z, 7Z, 10Z, 13Z, 16Z)/0:0), LysoPC (18:1(11Z)/0:0), LysoPC (20:4(5Z, 8Z, 11Z, 14Z)/0:0), and LysoPC (16:1(9Z)/0:0) had a significant negative correlation with Bacteroidetes (*p* < 0.05). Firmicutes had a significant positive correlation with LysoPC (15:5/0:0), LysoPC (18:1(11Z)/0:0), and PC (18:1(6Z)/0:0) (*p* < 0.05). *Faecalibaculum* had a significant positive correlation with PC (18:3/18:1), LysoPC (22:5(4Z, 7Z, 10Z, 13Z, 16Z)/0:0), and LysoPC (15:0/0:0) (*p* < 0.05). *Blautia* had a significant negative correlation with phenylalanine (*p* < 0.05), while *Allobaculum* had a significant positive correlation with LysoPC (18:1(11Z)/0:0) and PC (18:1(6Z)/0:0) (*p* < 0.05) (see Table 2).

**Table 2:** Correlations between intestinal microbiota, metabolic characteristics, and metabolites.

Intestinal microbiota	Metabolic characteristics	Correlations with liver metabolites	Significance
Firmicutes	Promotes energy absorption → Heat accumulation → Obesity	Significant positive correlation with LysoPC (15:5/0:0), LysoPC(18:1(11Z)/0:0), PC(18:1(6Z)/0:0)	*p* < 0.05
Bacteroidetes	Degrades polysaccharides and regulates host metabolism	Significant negative correlation with, LysoPC(22:5(4Z, 7Z, 10Z, 13Z, 16Z)/0:0), LysoPC (18:1(11Z)/0:0), LysoPC (20:4(5Z, 8Z, 11Z, 14Z)/0:0), LysoPC (16:1(9Z)/0:0)	*p* < 0.05
*Allobaculum*	Lactic acid-producing pathogenic bacteria; significantly enriched in high-fat diet (HFD)	significant positive correlation with LysoPC (16:1(9Z)/0:0), LysoPC (18:1(11Z)/0:0), PC (18:1(6Z)/0:0)	*p* < 0.05
*Faecalibaculum*	Pro-inflammatory bacteria → Damages intestinal barrier and exacerbates hepatic steatosis	significant positive correlation with PC (18:3/18:1), LysoPC (22:5(4Z, 7Z, 10Z, 13Z, 16Z)/0:0, LysoPC (15:0/0:0)	*p* < 0.05

### 3.9 Effect of RFV on the protein expression of AMPK signaling pathway in NAFLD mice

To further investigate the effect of RFV on the AMPK/FASN/SREBP-1c signaling pathway, Western blotting was performed to measure the expression levels of AMPK, FASN, and SREBP-1c in the liver.

**Figure 7:**
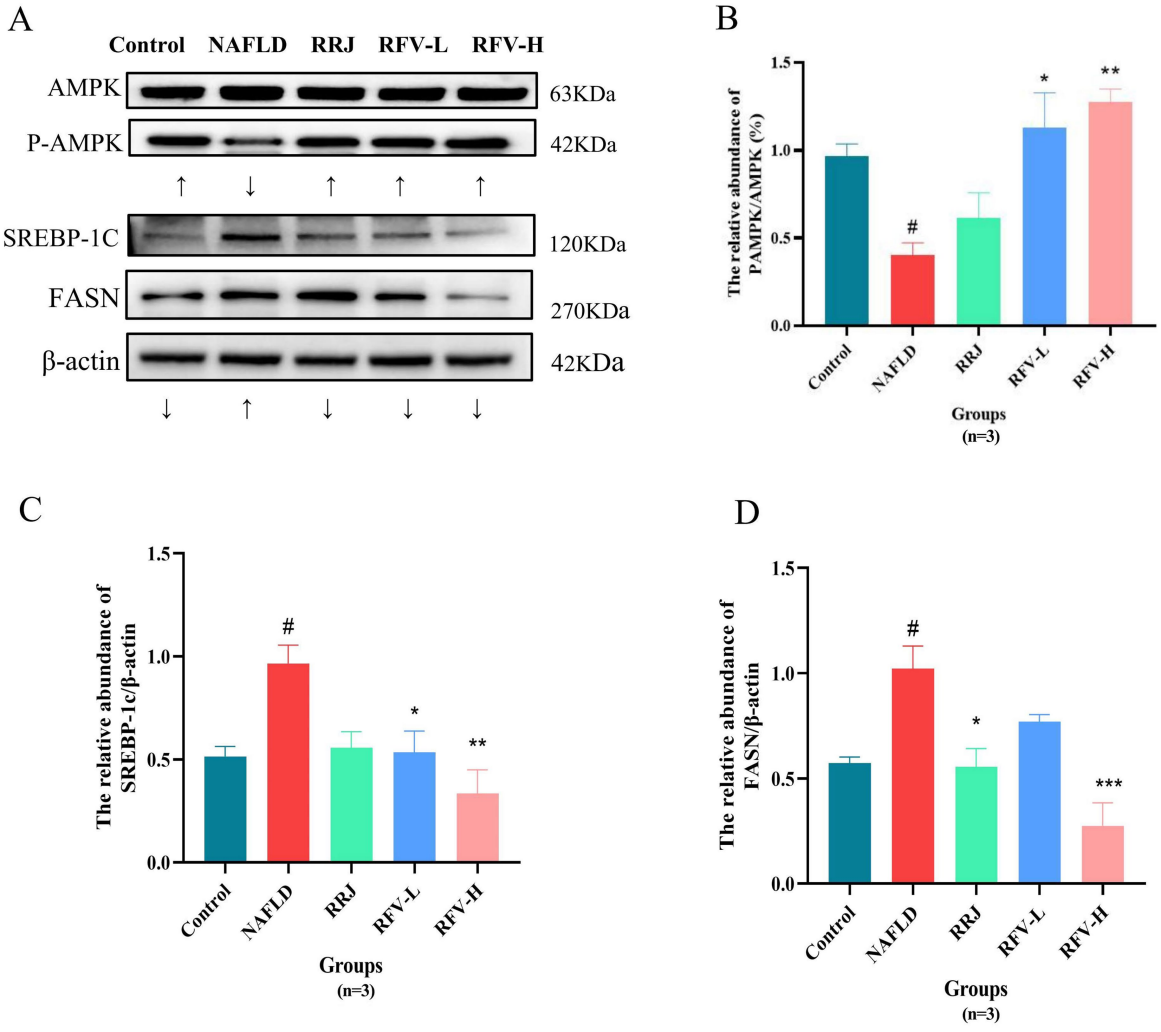
The protein expression and relative abundance of in liver. Protein bands diagram **(A)**; the gray values of P-AMPK/AMPK **(B)**; SREBBP-1c/β -actin **(C)**; FASN/β -actin **(D)**. #*p*<0.05, compared with Control group, *p<0.05, compared with NAFLD group (*n*=3).

Western blotting results demonstrated that compared to the Control group, the expression of P-AMPK in the NAFLD group (*n* = 3) was significantly inhibited (*p* < 0.05), whereas the expressions of SREBP-1c and FASN were significantly increased (*p* < 0.05) (Figure 7). After administration of RFV, AMPK expression was activated, and P-AMPK was significantly increased (*p* < 0.05, *p* < 0.01). Conversely, SREBP-1c and FASN expression levels were significantly reduced. In summary, RFV inhibited the expression of SREBP-1c and FASN by activating P-AMPK in the AMPK signaling pathway, thereby reducing lipid accumulation and improving NAFLD.

## 4 Discussion

NAFLD is a liver metabolic syndrome caused by obesity. Previous studies have demonstrated that RFV improves obesity and its related complications. This effect was achieved by regulating the intestinal microbiota of mice fed a high-fat diet (20). Consequently, a NAFLD mouse model was established using a high-fat diet. The mechanism by which RFV ameliorates NAFLD was further investigated.

The chemical components of RFV are highly diverse. However, the mechanisms by which RFV regulates NAFLD remain unclear. To address this, UPLC-MS was used to characterize the major constituents of RFV, and 70 compounds were identified (32). Based on the compounds identified in RFV, this study used network pharmacology and molecular docking to predict the potential targets and pathways through which RFV might ameliorate NAFLD. These results indicate that RFV may exert its ameliorative effects on NAFLD through these key compounds, including ellagic acid, gallic acid, bayogenin, asiatic acid, *α*-linolenic acid, and quercetin. Among these, quercetin, ellagic acid, and gallic acid can suppress hepatic lipogenesis and improve hepatic steatosis (35–37). This was achieved by regulating the expression of proteins involved in the AMPK signaling pathway. Based on NAFLD pathogenesis, including lipid accumulation, inflammatory factors, and insulin resistance and its progression (such as obesity, diabetes, liver disease, and cancer) (38), the following 11 relevant pathways were selected: lipid and atherosclerosis pathway, AGE-RAGE signaling pathway, AMPK pathway, C-type lectin receptor pathway, insulin resistance, HIF − 1 signaling pathway, toll-like receptor signaling pathway, TNF signaling pathway, alcoholic liver disease, NAFLD, and pathways in cancer. They regulate the oxidative stress, inflammatory response, and lipid metabolic disorders (39, 40). Furthermore, the HIF-1, toll-like receptor, and TNF signaling pathways are inflammatory pathways that regulate the expression of inflammatory factors such as TNF-α, IL-6, and IL-1β (41, 42). In summary, RFV may improve NAFLD by regulating lipid synthesis via the AMPK pathway.

Numerous studies have demonstrated that the intestinal microbiota and its metabolites influence the occurrence and progression of NAFLD through various mechanisms. To clarify whether the regulation of RFV in NAFLD mice led to changes in intestinal microbiota, the influence of a high-fat diet and RFV on the intestinal microbiota in mice was observed (43). At the phylum level, Firmicutes and Bacteroidetes were the dominant genera, and Firmicutes can produce energy for the body to absorb heat and promote obesity (44). Bacteroidetes play an important role in host metabolism, including the absorption and degradation of polysaccharides (45). F/B is associated with the susceptibility of obesity disease states (45), and F/B can be used an index symbolized as the disorder of metabolic diseases (46). A study reported that the abundance of Firmicutes and the ratio of F/B increased in obese individuals (47). At the genus level, the dominant genera in the Control group were *Lactobacillus* and *g_norank_f_Oscillospiraceae* and in the NAFLD group were *Faecalibaculum*, *Blautia*, *Allobaculum*, *Dubosiella*, *Blautia*, and *unclassified_f_Lachnospiraceae. Faecalibaculum* has been reported to be positively associated with serum lipids and the development of NAFLD (48). *Allobaculum* is a conditioned pathogen that produces lactic acid, which was significantly higher in the HFD group (49). It has been reported that the flora of the *Lachnospiraceae* family is typically associated with weight gain in obese mice (50). In contrast, *Blautia* is associated with the development of glucose metabolism disorders and is significantly positively correlated with obesity, inflammation, intestinal permeability, and metabolic endotoxemia (51, 52).

Compared to the NAFLD group, RFV-H significantly increased the relative abundance of these bacteria, with *Psychrobacter* being the dominant species. *Psychrobacter* is a beneficial bacterium. It can co-regulate disturbances in intestinal microbiota with other bacteria in high-fat diet-induced type 2 diabetes (53). Numerous studies have revealed that the abundance of *Akkermansia* is negatively correlated with body weight and obesity in mice and humans (54). In our study, compared to the normal-fed mice (Control group), the relative abundance of *Akkermansia* increased in high-fat diet-fed mice (NAFLD group) and was higher than in the Control group after intervention with RFV-L and RFV-H, but still lower than that in the NAFLD group. In a study of the effects of Shenling Baizhu Powder on intestinal microbiota in NAFLD mice, the relative abundance of *Akkermansia* in NAFLD mice and various administration groups was consistent with the results of this study (55). Finally, the correlation analysis revealed that the above bacteria were significantly positively correlated with TC, LDL-C, and MDA and significantly negatively correlated with SOD. Therefore, we speculate that the effects of RFV on liver lipid, liver function impairment, and oxidative stress in NAFLD may be related to regulating the relative abundance of Bacteroidetes, Firmicutes, *Allobaculum*, *Faecalibaculum*, *Dubosiella*, *Blautia*, and *unclassified_f_Lachnospiraceae.*

The glycerophospholipid metabolic pathway related to lipid metabolism may be a potential target for RFV intervention in NAFLD. Diacylglycerol produced by TG metabolism is used as a raw material to continue the production of PC or PE in the glycerophospholipid metabolic pathway, and PC can further produce LysoPC (56). Lipid accumulation due to TG is one of the leading causes of NAFLD, which in turn affects the metabolism of PC and PE. Some studies have reported that the plasma levels of PC and PE were disturbed in patients with NAFLD, and the levels of PC and PE in the livers of mice with NAFLD induced by a high-fat diet were significantly increased (57). LysoPC, the main component of LDL-C, induces lipid peroxidation of the cell membrane and leads to inflammation. LysoPC plays an important role in cell membrane processes and mediates signal transduction (58). Moreover, previous studies have demonstrated that PC and LysoPC levels are associated with fat decay, inflammation, and oxidative stress in hepatocytes (59, 60). The results of this study indicate that a high-fat diet increased the content of some metabolites prefixed with LysoPC and PC in the liver. However, the contents of these metabolites decreased significantly and exhibited a trend toward normal levels after RFV-H administration. These results indicated that RFV could alleviate the occurrence and development of NAFLD by regulating the expression of metabolites related to lipid accumulation and energy metabolism in glycerophospholipid metabolism. The correlation analysis (Figure 7D) revealed a significant interaction between intestinal microbiota and liver metabolites. Specifically, the abundance of phosphatidylcholine (PC) and lysophospholipid (LPC) metabolites was significantly associated with intestinal microbiota dysbiosis, which was characterized by an increased relative abundance of Firmicutes and a decreased of Bacteroidetes. Notably, Firmicutes showed significant positive correlations with PC (22:5/0:0), LysoPC (18:1(11Z)/0:0), and PC (18:1(6Z)/0:0). Bacteroidetes showed significant negative correlations with LysoPC (22:5(4Z,7Z,10Z,13Z,16Z)/0:0), LysoPC (18:1(11Z)/0:0), LysoPC (20:4(5Z,8Z,11Z,14Z)/0:0), and LysoPC (16:1(9Z)/0:0). Our research findings indicate that RFV mitigates NAFLD through intestinal microbiota–metabolite axis. Importantly, alterations in the intestinal microbiota act as the driving force influencing hepatoprotective metabolites, thereby ultimately reducing steatosis and oxidative stress.

Through non-targeted metabolomics, it was revealed that RFV may mitigate lipid metabolism dysregulation in NAFLD mice by modulating the glycerophospholipid metabolism pathway (Figures 5A,B). Importantly, metabolites such as phosphatidylcholines (PC) and lysophosphatidylcholine (LPC) within lipid metabolism pathways were significantly influenced by RFV (Figure 5D). These metabolites or derivatives of gut microbial metabolites served as energy substrates for intestinal and hepatic tissues while concurrently enhancing intestinal secretory functions and lipid metabolism (61, 62). From an integrated metabolic perspective, lipid metabolism constitutes a principal mechanism through which RFV ameliorates NAFLD-associated dysregulation. Nevertheless, the molecular signaling pathways by which these metabolites induce lipid metabolic disorders remain to be elucidated.

In the glycerophospholipid metabolic pathway, differential metabolites primarily participate in fatty acid synthesis, oxidation, and degradation. They are directly regulated by PPARα. On the one hand, the activity of PPARα is regulated by adenosine 5′-monophosphate (AMP)-activated protein kinase (AMPK) (63). AMPK activation inhibits downstream targets fatty acid synthase (FASN) and sterol element regulatory protein (SREBP-1c), regulating hepatic lipid metabolism disorders (64, 65). On the other hand, based on network pharmacology and literature analysis, the active compounds in RFV may improve NAFLD. These compounds could activate the AMPK/SREBP-1c/FASN lipid synthesis pathway and reduce lipid accumulation (66). Therefore, we proposed that RFV ameliorated lipid metabolism dysregulation via the AMPK signaling pathway. Then, further analysis of the protein expression levels of FASN and SREBP-1C in lipid synthesis confirmed the results of this study. RFV was observed to activate AMPK phosphorylation and reduce the expression levels of FASN and SREBP-1c (Figure 7). The mechanism by which RFV improves lipid metabolism disorders in high-fat diet mice is by activating the AMPK pathway, regulating the expression of downstream target proteins, weakening fatty acid synthesis, and promoting fatty acid breakdown.

This study demonstrated RFV-mediated regulation of intestinal microbiota via 16S rRNA analysis and predicted multi-target effects against NAFLD through network pharmacology. As a functional food, RFV has been proven to be effective in improving NAFLD. Nonetheless, the investigation did not address the impact on the intestinal acid–base environment and the milieu of intestinal epithelial cells, thereby neglecting potential effects on essential functions such as nutrient absorption efficiency, gut barrier integrity, and mucosal immune responses. The experimental validation was confined to the AMPK signaling pathway and did not encompass the other prediction pathways, such as insulin resistance and PPARα. The real-time luminal pH mapping coupled with single-cell transcriptomic profiling of the intestinal epithelium can be integrated into future studies. This dual-axis approach will provide insights into how acid–base dynamics influence the gut–microbe–immune interface, particularly through pH-sensitive receptors such as GPR65 and electrolyte transporters such as NHE3. In addition, it is essential to detect the expression levels of intestinal epithelial tight junction proteins, such as Occludin, Claudins, and ZO-1, to further elucidate the mechanisms underlying the “gut–liver” axis. Insulin signaling and PPARα pathways will be validated to elucidate multi-target mechanisms of RFV.

In conclusion, as a functional food, RFV has been proven to be effective in improving NAFLD. The underlying mechanisms involve the modulation of the intestinal microbiota and metabolites balance and regulation on lipid disorders through AMPK signaling pathway. These findings not only furnish a theoretical foundation for the development and application of RFV but also propose a novel approach for the improvement of NAFLD by dietary therapy.

## Data Availability

The datasets presented in this study can be found in online repositories. The names of the repository/repositories and accession number(s) can be found in the article/Supplementary material.

## Footnotes

^1^ http://www.swisstargetprediction.ch/

^2^ https://bioinfogp.cnb.csic.es/tools/venny/

^3^ https://cn.string-db.org/

^4^ https://david.ncifcrf.gov/

^5^ https://www.bioinformatics.com.cn/

^6^ https://pubchem.ncbi.nlm.nih.gov

## Glossary

NAFLD: non-alcoholic fatty liver disease
NASH: non-alcoholic steatohepatitis
RRJ: *Rosa Roxburghii* Tratt. juice
RFV: *Rosa Roxburghii* Tratt. fruit vinegar
RFV-L: low-dose RFV (group)
RFV-H: high-dose RFV (group)
GO: Gene Ontology
KEGG: Kyoto Encyclopedia of Genes and Genomes
OTUs: operational taxonomic units
F/B: Firmicutes to Bacteroidetes
SOD: superoxide dismutase
TG: triglycerides
TC: total cholesterol
MDA: malondialdehyde
HDL-C: high-density lipoprotein
LDL-C: low-density lipoprotein
PLS-DA: partial least squares discriminant analysis
PA: phosphatidic acid
PC: phosphatidylcholine
GPC: glycerophosphate
LysoPC: lysophosphatidylcholine
PS: phosphatidylserine
AMPK: phosphorylation of AMP-activated protein kinase
FASN: fatty acid synthase
SREBP-1c: sterol regulatory element-binding protein 1
